# Association of alpha-fetoprotein and metastasis for small hepatocellular carcinoma: A propensity-matched analysis

**DOI:** 10.1038/s41598-022-19531-8

**Published:** 2022-09-20

**Authors:** Yu-Qi Wang, An-Jiang Wang, Ting-Ting Zhang, Si-Hai Chen

**Affiliations:** 1 Department of Gastroenterology, The First Affiliated Hospital of Nanchang University, Nanchang, 330006 China; 2 Department of Gastroenterology and Hepatology, Shenzhen Hospital, Southern Medical University, Shenzhen, China

**Keywords:** Cancer, Medical research

## Abstract

Metastasis is crucial for the prognosis of hepatocellular carcinoma (HCC). Distinguishing the potential risk factors for distant metastasis in small HCC (diameter ≤ 5 cm) is of great significance for improving the prognosis. HCC patients in the Surveillance, Epidemiology and End Results (SEER) registry with tumors ≤ 5 cm in diameter between January 2010 and December 2015 were retrieved. Demographic and clinicopathological metrics were extracted, including age, sex, race, marital status, tumor size, histological grade, T stage, N stage, M stage, alpha-fetoprotein (AFP), and liver fibrosis score. Univariate and multivariate logistic regression analyses were used to identify independent risk factors correlated with extrahepatic metastasis in small HCC. Propensity score matching (PSM) analysis was performed to balance the confounding factors in baseline characteristics. A total of 4176 eligible patients were divided into a non-metastasis group (n = 4033) and a metastasis group (n = 143) based on metastasis status. In multivariate analysis, larger tumor size, poor histological differentiation, regional lymph node metastasis, and elevated serum AFP levels were identified as independent risk factors for distant metastasis (*P* < 0.05), while age, sex, race, marital status, and liver fibrosis score were not associated with extrahepatic metastasis. After propensity score analysis, the AFP level was no longer associated with metastatic risk. The present study provided no evidence for a correlation between the clinical threshold of AFP and metastasis in small hepatocellular carcinoma.

## Introduction

Liver cancer is currently the sixth most frequently diagnosed neoplasm and the fourth leading cause of cancer-related mortality worldwide, with approximately 841,000 new cases and 782,000 deaths in 2018^1^. Hepatocellular carcinoma (HCC), as the major histological type of primary liver cancer, accounts for approximately 90% of all cases^2^. Although advances in therapeutic strategies, such as liver transplantation, surgical resection, radiofrequency ablation (RFA), and transarterial chemoembolization (TACE), have greatly improved the prognosis of HCC^3–6^, the 5-year survival rate remains below 20%^7,8^. Metastasis accounts for a great proportion of cancer-related deaths; approximately 90% of deaths are caused by metastatic disease rather than primary tumors, and limited treatment options can be applied for extrahepatic metastasis^9^.

The majority of HCC cases are in middle or advanced stages at the time of diagnosis beyond the curative therapeutic regimens^9–11^. HCC diagnosed at early stages could be cured by liver transplantation and liver resection, which makes the 5-year survival over 50% possible^10,12^. Although liver transplantation is the best treatment option for those who are not suitable for surgical resection, it is limited due to donor shortage; therefore, liver resection remains the major curative treatment strategy in clinical practice^10^. A complete response for HCC with a diameter < 5 cm (small HCC) could be achieved after surgical resection, but the recurrence rate for small HCC is still high due to various contributing factors in recurrence and metastasis^11,13^. Accordingly, identifying small HCC patients with a high risk of extrahepatic metastasis is meaningful in clinical practice.

Previous studies had indicated that some clinicopathological factors can were risk factors of metastasis in HCC patients. Several studies reported that tumor size, vascular invasion, histological differentiation, tumor stage, serum AFP, etc., increased the risk for distant metastasis^14–16^. Other general characteristics of patients, including age, sex, race, etc., also indicate a certain role in the prognosis of HCC^17^. In addition, a growing body of literature had investigated the role of gene expression on HCC progression and metastasis. Simultaneously, gene expression was found to be associated with these tumor characteristics^18,19^. Thus, clinicopathological factors exert an important role in the estimation of HCC metastasis.

Although several studies have explored the association between tumor size and extrahepatic metastatic risk in HCC, for HCC tumors less than 5 cm in diameter, as far as we know, there are limited data to fully clarify the relationship. Besides, there is no research that focused on whether AFP is the risk factor for patients with small HCC.

In the present study, we retrospectively reviewed the data from the Surveillance, Epidemiology, and End Results (SEER) database to investigate the potential risk factors and AFP related to extrahepatic metastasis in small HCC (diameter ≤ 5 cm).

## Results

### Clinicopathological characteristics of the patients

A total of 4176 HCC patients were eligible for analysis. Of these, 143 cases (3.4%) presented with distant metastasis, while the remaining 4033 patients (96.6%) did not. The available clinicopathological metrics and their differences between the two groups are summarized in Table 1. Compared to the non-metastasis group, the metastasis group had significantly larger tumor diameters on average, higher levels in histological grade and T stage, greater likelihood of being unmarried at diagnosis and regional lymph node metastasis, with a higher proportion of elevated AFP levels but a lower degree of liver fibrosis score, and with no significant differences in race, sex and age at diagnosis between groups.

**Table 1:** Clinicopathological characteristics of patients with small HCC and their differences between distant metastasis and non-distant metastasis groups.

Variable	Total cohort (n = 4176)	Non-metastasis (n = 4033)	Metastasis (n = 143)	*P* value
Age, mean ± SD, year	63.6 ± 9.9	63.6 ± 9.8	63.5 ± 12.0	0.944
**Gender, n (%)**				
Male	3084 (73.9)	2970 (73.6)	114 (79.7)	0.104
Female	1092 (26.1)	1063 (26.4)	29 (20.3)	
**Race, n (%)**				
Black	568 (13.6)	542 (13.4)	28 (19.6)	0.059
White	2822 (67.6)	2727 (67.6)	95 (66.4)	
Others*	784 (18.8)	764 (18.9)	20 (14.0)	
**Marital status, n (%)**				
Married	2414(57.8)	2346 (58.2)	68 (47.6)	**0.012**
Unmarried	1762(42.2)	1687 (41.8)	75 (52.4)	
Tumor size, mean ± SD, mm	30.5 ± 10.9	30.4 ± 10.9	33.9 ± 12.2	** < 0.001**
**Histological grade, n (%)**				
I	1421 (34.0)	1382 (34.3)	39 (27.3)	** < 0.001**
II	2084 (49.9)	2036 (50.5)	48 (33.6)	
III + IV	671 (16.1)	615 (15.2)	56 (39.2)	
**T stage, n (%)**				
T1	2483(59.5)	2436 (60.4)	47 (32.9)	** < 0.001**
T2	1693(40.5)	1597 (39.6)	96 (67.1)	
**N stage, n (%)**				
N0	4068 (97.4)	3964 (98.3)	104 (72.7)	** < 0.001**
N1	108 (2.6)	69 (1.7)	39 (27.3)	
**AFP, n (%)**				
Negative	1602 (38.4)	1570 (38.9)	32 (22.4)	** < 0.001**
Positive	2574 (61.6)	2463 (61.1)	111 (77.6)	
**Fibrosis score, n (%)**				
F0 (0–4)	385 (9.2)	374 (9.3)	11 (7.7)	**0.001**
F1 (5–6)	1379 (33.0)	1351 (33.5)	28 (19.6)	
Unknown	2412 (57.8)	2308 (57.2)	104 (72.7)	

Significant values are in bold.

*The others comprise American Indian/Alaska Native, Asian/Pacific Islander. *AFP* alpha-fetoprotein, *HCC* hepatocellular carcinoma.

### Risk factors for the distant metastasis of small HCC

Upon univariate analysis, seven factors (race, marital status, tumor size, histological grade, T stage, N stage and AFP level) were significantly related to extrahepatic metastasis. From the results of multivariate analysis, it was found that a larger tumor size, poorer histological differentiation, regional lymph node metastasis, and elevated AFP levels were significantly correlated with distant metastasis risk in small HCC (Table 2).

**Table 2:** Associations of clinicopathological metrics with distant metastasis in small HCC patients.

Variable	Univariate logistic analysis		Multivariate logistic analysis	
	OR (95%CI)	*P* value	OR (95%CI)	*P* value
Age	0.999 (0.983–1.016)	0.944		
**Gender**				
Male	Reference			
Female	0.711 (0.470–1.075)	0.106		
**Race**				
Black	Reference			
White	0.674 (0.438–1.038)	0.073		
Others*	0.507 (0.283–0.909)	0.023		
**Marital status**				
Married	Reference			
Unmarried	1.534 (1.098–2.142)	0.012		
Tumor size	1.031 (1.015- 1.047)	< 0.001	1.024 (1.007–1.041)	0.005
**Histological grade**				
I	Reference		Reference	
II	0.835 (0.545–1.282)	0.410	0.792 (0.508–1.235)	0.303
III + IV	3.227 (2.121–4.909)	< 0.001	2.346 (1.492–3.688)	< 0.001
**T stage**				
T1	Reference			
T2	3.116 (2.186–4.442)	< 0.001		
**N stage**				
N0	Reference		Reference	
N1	21.543 (13.899–33.393)	< 0.001	16.015 (10.143–25.287)	< 0.001
**AFP**				
Negative	Reference		Reference	
Positive	2.211 (1.485–3.293)	< 0.001	1.679 (1.101–2.563)	0.016
**Fibrosis score**				
F0 (0–4)	Reference			
F1 (5–6)	0.705 (0.348–1.429)	0.332		
Unknown	1.532 (0.815–2.879)	0.185		

*The others comprise American Indian/Alaska Native, Asian/Pacific Islander. *AFP* alpha-fetoprotein, *HCC* hepatocellular carcinoma, *OR* odds ratio, *CI* confidence interval.

### Confirmation of AFP as a risk factor for small HCC metastasis by PSM

**Figure 1:**
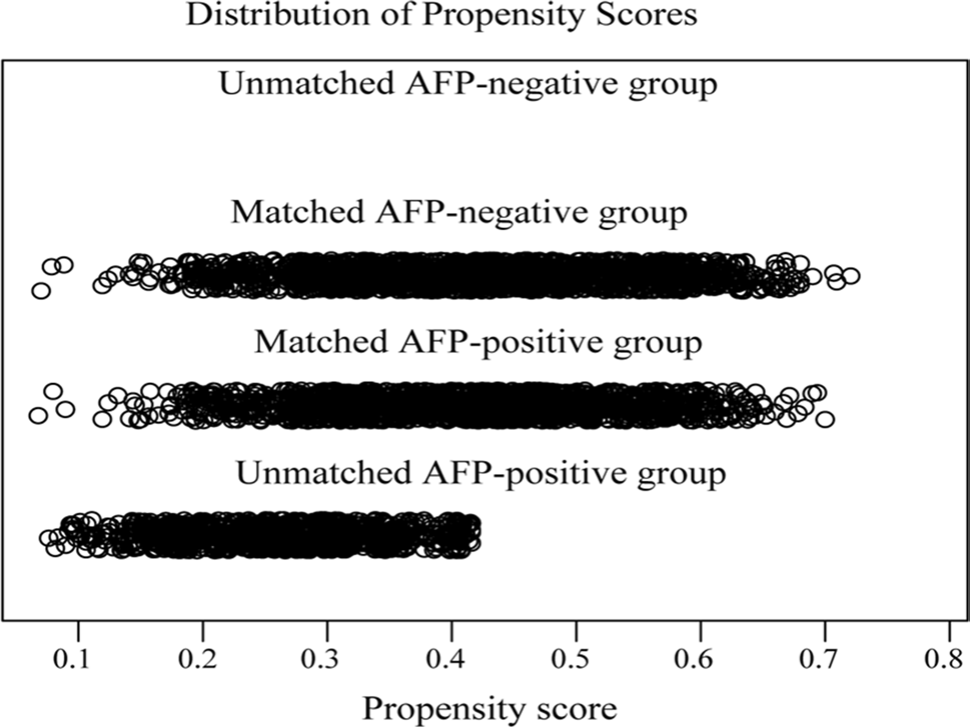
Distribution of propensity scores before and after matching. The distribution of propensity scores of matched patients between groups were similar.

**Figure 2:**
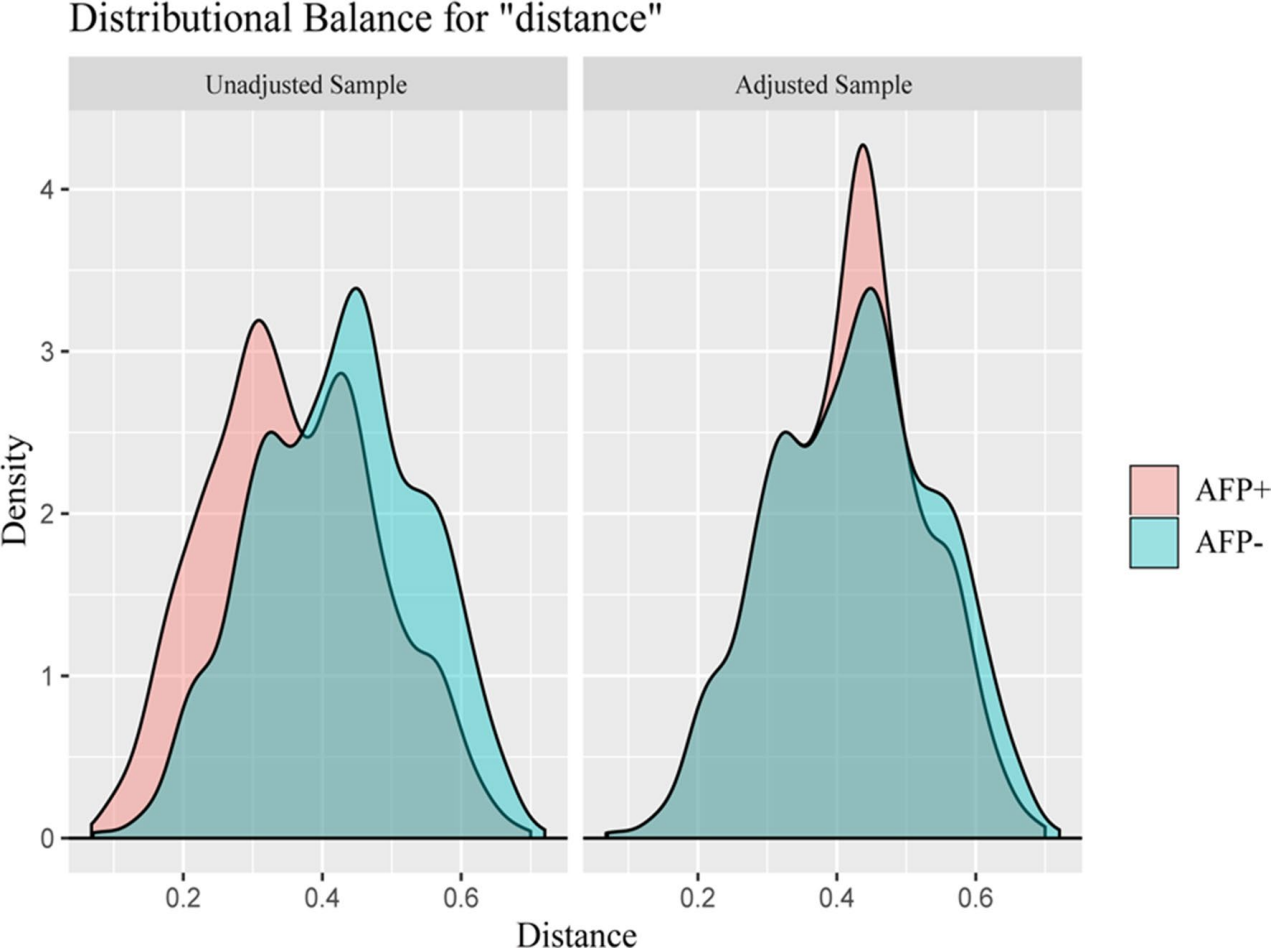
Overlay of density distributions of AFP-positive and AFP-negative groups of propensity scores before and after propensity score matching.

We found that the AFP level was one of four significant risk factors in the multivariate analysis. However, the value of AFP in HCC diagnosis, especially for small ones, and its ability to predict extrahepatic metastasis remain uncertain and inconsistent. Due to the imbalance in variable distributions, comparison between groups would be confounded by selection bias. To minimize the confounding impacts, PSM was performed between the AFP-negative and AFP-positive groups and resulted in 2124 patients (1062 in each group) being matched (Table 3). After matching the sample in terms of characteristics, including age, sex, race, marital status, tumor size, T stage, N stage, and fibrosis score, none of the covariates differed significantly (all *P* > 0.05), except for race and fibrosis score (*P* < 0.05). The distributions of propensity scores between the two groups were similar in the jitter plot (Fig. 1), and the distribution balance for AFP before and after PSM illustrated a good match between groups after PSM (Fig. 2). In the propensity score matched small HCC patients, serum AFP positivity was not identified as an independent determinant of extrahepatic metastasis, with an OR of 1.45 (95% CI 0.919–2.29, *P* = 0.11) compared with AFP negativity. Thus, the AFP level may not be associated with metastatic risk estimation for small HCC.

**Table 3:** Comparison of clinicopathological metrics between the AFP positive and negative groups in 2124 small HCC patients matched by propensity scores.

Variable	AFP (−) (n = 1062)	AFP (+) (n = 1062)	*P* value
Age, (mean ± SD), year	64.3 ± 10.3	63.9 ± 9.5	0.265
**Gender, n (%)**			
Male	1239 (77.3)	1216 (75.9)	0.337
Female	363 (22.7)	386 (24.1)	
**Race, n (%)**			
Black	142 (8.9)	195 (12.2)	**0.002**
White	1143 (71.3)	1063 (66.4)	
Others*	317 (19.8)	344 (21.5)	
**Marital status, n (%)**			
Married	986 (61.5)	961 (60.0)	0.366
Unmarried	616 (38.5)	641 (40.0)	
Tumor size, mean ± SD, (mm)	30.0 ± 11.2	29.6 ± 11.0	0.881
**Histological grade, n (%)**			
I	675 (42.1)	642 (40.1)	0.495
II	778 (48.6)	805 (50.2)	
III + IV	149 (9.3)	155 (9.7)	
**T stage, n (%)**			
T1	1095 (68.4)	1066 (66.5)	0.274
T2	507 (31.6)	536 (33.5)	
**N stage, n (%)**			
N0	1579 (98.6)	1579 (98.6)	1.000
N1	23 (1.4)	23 (1.4)	
**Fibrosis score, n (%)**			
F0 (0–4)	171 (10.7)	136 (8.5)	**0.021**
F1 (5–6)	502 (31.3)	562 (35.1)	
Unknown	929 (58.0)	904 (56.4)	
**Metastatic status, n (%)**			
Negative	1570 (98.0)	1556 (97.1)	0.109
Positive	32 (2.0)	46 (2.9)	

Significant values are in bold.

*The others comprise American Indian/Alaska Native, Asian/Pacific Islander. *AFP* alpha-fetoprotein, *HCC* hepatocellular carcinoma.

## Discussion

In the current study, we collected registry information for small HCC from the SEER database, and the multivariate analysis revealed that tumor size, histological grade, N stage, and AFP level were independent risk factors for extrahepatic metastasis in small HCC. The relationship between metastatic risk and the above variables of tumor size, histological grade, and N stage are definite, while the impact of AFP level on metastatic risk remains uncertain. To further validate the association between AFP and extrahepatic metastasis, the AFP-positive group was matched to the AFP-negative group to reduce the effects of selection bias. After PSM, the AFP level was statistically insignificant.

Tumor size was identified as an important factor for extrahepatic metastasis in several previous studies. Wu et al.^8^ reviewed the data of 33,177 HCC patients from the SEER database and indicated that a maximum tumor size over 5 cm was related to a higher risk of lung metastasis. Similarly, Yokoo et al.^14^ conducted a retrospective case–control study on 236 newly diagnosed HCC and found that tumor size > 5 cm was independently associated with metastasis risk but with moderate sensitivities of 75.6% (69.6–80.7%) in the screening test. In addition, an observation study that enrolled 1573 HCC patients revealed that tumor diameter > 2 cm had a significantly higher risk of extrahepatic metastasis compared to those tumors < 2 cm^20^. In our present study, we also observed that a larger tumor diameter within 5 cm also had a higher risk of metastasis. Consequently, HCC with tumors less than 5 cm in diameter also has a higher risk of metastasis.

Histological differentiation grade and N stage (lymph node metastasis) are important characteristics of malignancy. Poorer histological differentiation and lymph node metastasis represent a higher degree of malignancy with poor prognosis. The two indicators have been extensively demonstrated to be associated with HCC metastasis. Lee et al. found that the overexpression of metastasis-associated protein MTA2 was detected in 96.2% of the 506 HCC tissue samples, which was tightly correlated with HCC size and differentiation^21^. Jia et al. reported that upregulated myeloid differentiation factor 88 (MyD88) may promote epithelial-mesenchymal transition (EMT) properties and tumor-initiating capabilities via the PI3K/Akt pathway, resulting in accelerated tumor growth and metastasis^22^. In addition, a large-scale study of 33,177 patients indicated that poor tumor pathological differentiation was a contributory factor to lung metastasis of HCC^8^, which is consistent with our results. In the present study, compared to well-differentiated HCC, poorly differentiated and undifferentiated HCC showed a significant tendency of metastasis, with the rationale being that poor tumor pathological differentiation was more likely to metastasize. Regarding lymph node metastasis, Bi et al.^23^ investigated the clinical relevance of MDM2 Binding Protein (MTBP) and found that MTBP was poorly expressed in HCC tissues compared to adjacent nontumor tissue, and its expression was negatively associated with lymph node metastasis and vascular invasion. Additionally, He et al.^24^ evaluated the clinical implications of another tumor suppressor of AT-rich interactive domain-containing protein 1A (ARID1A) and found that decreased ARID1A levels were significantly associated with regional lymph node and distant metastasis. Many similar studies indirectly suggested that lymph node metastasis was a poor prognostic factor for HCC.

AFP has been extensively applied as a biomarker for HCC diagnosis clinically. However, recent data have shown that the low sensitivity and specificity are challenges in the diagnosis of HCC^25^, especially for early stage HCC, as the sensitivity of combined serum AFP and ultrasonography only varies from 40 to 65%^26^. Accordingly, the value of serum AFP to predict metastasis in small HCC remains to be explored. Jin et al.^27^ detected the AFP mRNA level in circulating tumor cells and found that the release of AFP from HCCs released into circulation could be a significant predictor for metastasis before and after hepatic resection. Yokoo et al.^14^ also indicated that AFP > 400 μg/mL was strongly correlated with extrahepatic metastasis. In contrast, Giannini et al.^28^ found that increased AFP levels did not show significance for prognosis in well-compensated cirrhosis patients with single, small HCC. In this study, the serum AFP level was identified as a risk factor before PSM, but while adjusting for confounding factors, the AFP level was not significantly associated with HCC metastasis with a diameter < 5 cm. One reason for the opposite results may be that clinical AFP detection is generally at the protein level, rarely at the RNA level. Another reason, perhaps owing to the existence of selection bias, is that the significance of AFP for metastasis may be affected by confounding covariates. Furthermore, our studies mainly focused on small HCC, while most similar studies took all diameters into consideration.

The main strength of this study is that we focused on small HCC with diameters of less than 5 cm; a better understanding of the significant determinants of extrahepatic metastasis in small HCC can help to reasonably manage tumors. In addition, the patients’ information was retrieved from a large population-based database (SEER). Furthermore, PSM was introduced to balance confounding factors in this observational study. Although traditional regression models have been widely used to adjust for confounding factors associated with exposure and outcome, the membership bias of characteristics between the treatment and control groups may not be adequately explained. Additionally, the results estimated with regression models may vary as the parameter settings change in certain circumstances^29–31^. PSM is able to balance the variable distribution so that the baseline characteristics between groups became comparable, thus reducing the potential influence of confounders and providing a more accurate estimation on causal effect results^32^. Thus, the present findings were more accurate and reliable.

However, the present study has several limitations. First, this is a retrospective study, although PSM analysis has the advantage of eliminating the confounding effects of observed covariates, the unmeasured factors may also play roles in causal effect estimation^33^. Second, the SEER database does not include other important risk factors, such as vascular invasion, family history, and genetic status. Therefore, more potential confounding factors should be evaluated in future research. Thirdly, the SEER database did not have the exact value of AFP, and just divided the value of AFP into positive and negative group based on the borderline. The continuous variables are converted into categorical form for analysis, which causes some information would lose^34^. Hence, the various cut-off value of AFP is needed to explore the relation between AFP level and metastasis of patients with small HCC.

## Conclusion

In summary, this study reveals that larger tumor size, poor tumor histological differentiation, and regional lymph node metastasis were associated with a higher risk of extrahepatic metastasis, but not AFP. Thus, this study provides a reference for small HCC patients with metastatic risk, and intensive attention should be paid to these patients.

## Methods

### The data source of patients

**Figure 3:**
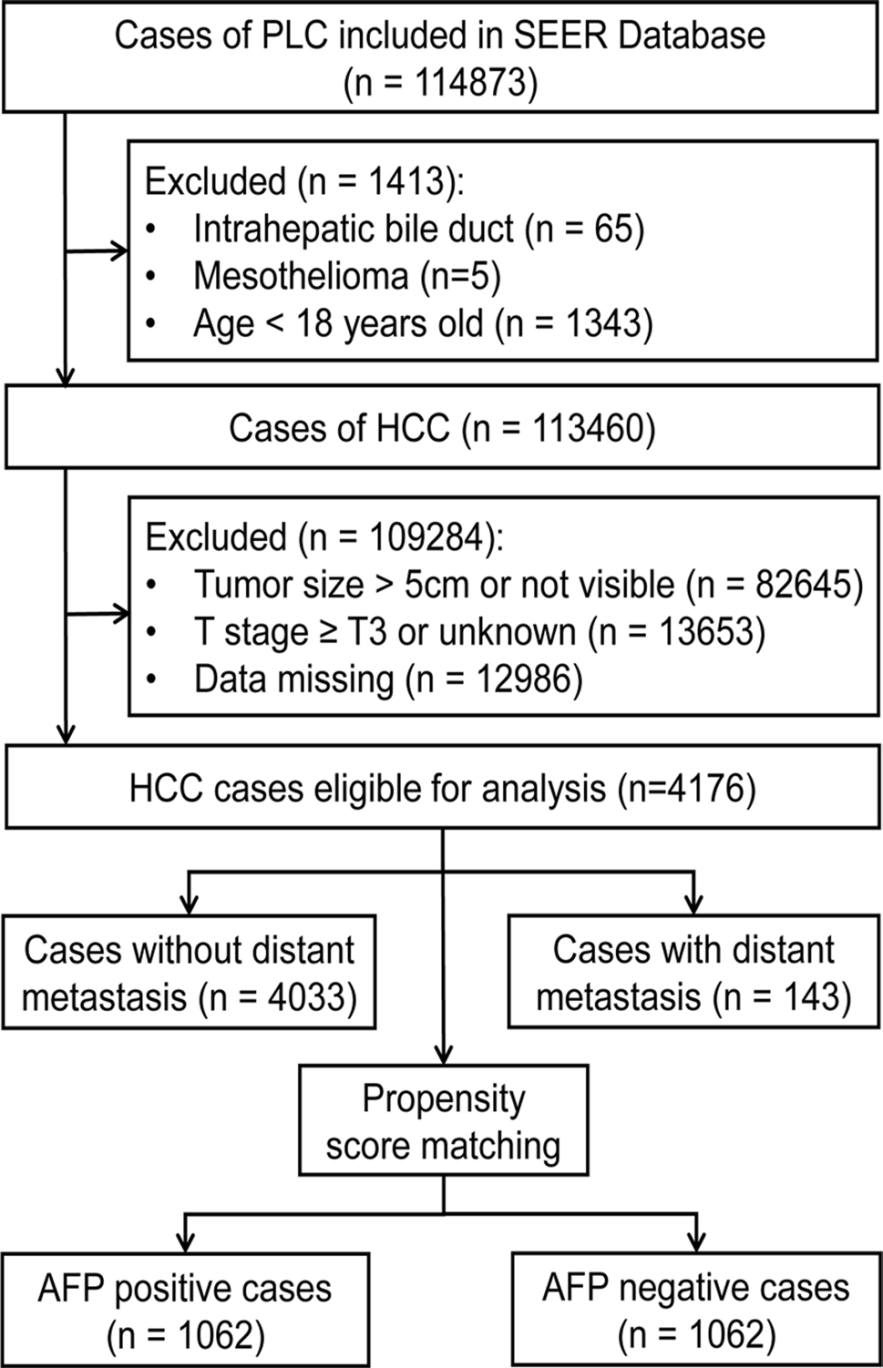
Flow chart of patient enrollment. *PLC* primary liver cancer, *SEER* the Surveillance, Epidemiology, and End Results (SEER) program of National Cancer Institute, *HCC* hepatocellular carcinoma.

Data of patients with primary liver cancer (PLC) were retrieved from the Surveillance, Epidemiology, and End Results (SEER) program of the National Cancer Institute, which is publicly available and provides access to cancer-related data from cancer statistic registries (1973–2015). A total of 114,873 patients with PLC were retrieved, and their clinicopathological metrics available were extracted from the database, including age at diagnosis, sex, race, marital status, alpha-fetoprotein (AFP), liver fibrosis score, tumor size, histological grade, and TNM stage (including explicit T, N, and M stages based on the 7th American Joint Committee on Cancer). The exclusion criteria for HCC patients are shown in Fig. 3.

### Data processing and statistical analysis

Univariate analysis and multivariate logistic regression analysis were applied to identify risk factors associated with extrahepatic metastasis in small HCC. Variables that were significant with *P* < 0.2 in univariate analysis were used in multivariate logistic regression for further analysis^35^. The significance of AFP as a risk factor was further confirmed after propensity score matching (PSM) in which the following covariates were matched: age, sex, race, marital status, histological grade, T stage, N stage, and fibrosis score. The matching algorithm of nearest neighbors was used in PSM. The R (3.5.2) package “MatchIt” was used to perform PSM, distribution of propensity scores were visualized with the generic plot() function, and propensity score analysis assessment plots of covariates were generated with the “cobalt” package.

In the statistical analysis, categorical variables are shown as numbers (%), and continuous variables are presented as the mean ± SD. Chi-square test or Fisher’s exact test was used to compare the differences for categorical variables, while continuous tests using Student’s t-test and Wilcoxon rank-sum test were applied when appropriate. Two-tailed *P* values < 0.05 were considered statistically significant in all analyses. Data were analyzed by SPSS for Windows version 22.0 (IBM, NY, USA) except for specific data described above.

## Data Availability

The datasets generated and analysed during the current study are available in the SEER repository. The data used in this study are available free of charge online at seer.cancer.gov on request. Data openly available in a public repository.
